# Genome-wide identification and expression analysis of calcium-dependent protein kinase in maize

**DOI:** 10.1186/1471-2164-14-433

**Published:** 2013-07-01

**Authors:** Xiangpei Kong, Wei Lv, Shanshan Jiang, Dan Zhang, Guohua Cai, Jiaowen Pan, Dequan Li

**Affiliations:** 1 State Key Laboratory of Crop Biology, Shandong Key Laboratory of Crop Biology, College of Life Sciences, Shandong Agricultural University, Tai’an, Shandong, 271018, China

**Keywords:** CDPK, Expression Analysis, Gene Family, Maize

## Abstract

**Background:**

Calcium-dependent protein kinases (CDPKs) have been shown to play important roles in various physiological processes, including plant growth and development, abiotic and biotic stress responses and plant hormone signaling in plants.

**Results:**

In this study, we performed a bioinformatics analysis of the entire maize genome and identified 40 CDPK genes. Phylogenetic analysis indicated that 40 ZmCPKs can be divided into four groups. Most maize CDPK genes exhibited different expression levels in different tissues and developmental stages. Twelve CDPK genes were selected to respond to various stimuli, including salt, drought and cold, as well as ABA and H_2_O_2_. Expression analyses suggested that maize CDPK genes are important components of maize development and multiple transduction pathways.

**Conclusion:**

Here, we present a genome-wide analysis of the CDPK gene family in maize for the first time, and this genomic analysis of maize CDPK genes provides the first step towards a functional study of this gene family in maize.

## Background

Plants consistently suffer from various environmental challenges, including drought, high salinity and low temperatures [1-3]. In response to these stresses, plants have developed a series of survival mechanisms. Among them, calcium (Ca^2+^), the second messenger in cells, plays an essential role in various signaling transduction pathways [4,5]. Transient changes in Ca^2+^ concentration are sensed by several Ca^2+^ sensors or Ca^2+^-binding proteins. To date, three major classes of Ca^2+^-binding proteins, including calcium dependent protein kinases (CDPK), calmodulins (CaM) and CaM-like proteins (CaML) and calcineurin B-like proteins (CBL), have been characterized in higher plants [6,7].

The CDPK are one of the well-known Ca^2+^-sensor protein kinases involved in environmental stress resistance, and these kinases are found in plants and some protozoans [8,9] but not in animals. The CDPK protein has four characterized domains: an *N*-terminal variable region, a Ser/Thr kinase catalytic domain, an autoregulatory/autoinhibitory domain and a calmodulin-like domain [10-12]. The calmodulin-like domain contains EF-hands for Ca^2+^ binding.

Accumulating evidence indicates that CDPKs play important roles not only in response to a broad variety of abiotic and biotic stresses, such as drought, cold, salinity, wounding and pathogen infection, but also in the signaling of plant hormones [13-21]. AtCPK4 and AtCPK11 are two positive regulators involved in CDPK/Ca^2+^-mediated ABA signaling through the phosphorylation of two ABA-responsive transcription factors, ABF1 and ABF4 [22]. AtCPK3 and AtCPK6 were shown to regulate guard cells ion channel activity and were shown to be involved in ABA-regulated stomatal signaling [23]. Moreover, plants over-expressing *AtCPK6* and *AtCPK3* showed enhanced tolerance to salt/drought stresses, whereas *atcpk6* mutant plants displayed no obvious phenotypes [24,25]. AtCPK6 also positively regulated methyl jasmonate (MeJA) signaling in guard cells [26]. *Atcpk21* and *atcpk23* mutants showed increased tolerance to hyperosmotic stress, drought and salt stresses [27,28]. *AtCPK32* overexpression enhanced ABA and salt sensitivities during germination through the phosphorylation of ABF4 [29]. *Arabidopsis cpk5*/*cpk6*, *cpk5*/*cpk6*/*cpk11* and *cpk5*/*cpk6*/*cpk11*/*cpk4* mutants compromised an flg22-induced response, including ROS production and defense-related gene expression [30]. In rice, the overexpression of *OsCDPK7* has been shown to enhance resistance to cold, drought and salt stress [31]. *OsCPK21* positively regulated ABA signaling and salt stress [32]. More recently, *OsCPK12* overexpression resulted in increased tolerance to salt stress and increased susceptibility to both compatible and incompatible blast fungi [33].

CDPKs are encoded by a large family. There are 34 CDPK genes in the *Arabidopsis* genome, 31 genes in rice and 20 genes in wheat [19,34-38]. Tobacco (*Nicotiana tabacum*), soybean (*Glycine max*) and tomato (*Lycopersicon esculentum*) also have multiple CDPK genes [39-43]. However, little is currently known about the CDPK family in maize. Maize (*Zea mays* L.) is one of the oldest and most important crops worldwide. So far, only seven ZmCPKs (*ZmCK1*, *ZmCDPK1*, *ZmCDPK2*, *ZmCDPK7*, *ZmCDPK9*, *ZmCDPK10* and *ZmCDPK11*) have been characterized in maize. It was reported that low temperatures induce *ZmCDPK1* expression in maize leaves [44]. The transcript levels of *ZmCDPK7* and *ZmCDPK9* were higher in roots and etiolated leaves than in green leaves, suggesting these two genes may be down-regulated in response to light [45]. *ZmCDPK10* expression occurred during the growth and development of the maize seedling in response to fungal infection and treatment with fungal elicitors [46]. Recently, the expression and enzymatic activity of ZmCDPK11 were shown to be regulated by linolenic acid (LA) and MeJA, and ZmCDPK11 participated in JA-dependent wound signaling pathways [21,47].

In this study, we performed bioinformatics analysis of the entire maize genome and identified 40 CDPK genes. These 40 maize CDPKs have been grouped based on their phylogenetic relationships and are anchored to specific chromosomes. The expression levels of twelve CDPK genes in maize roots were measured to assess the responses to cold, drought, salt, ABA and H_2_O_2._

## Results and discussion

### Genome-wide identification of ZmCPK gene family members

**Figure 1:**
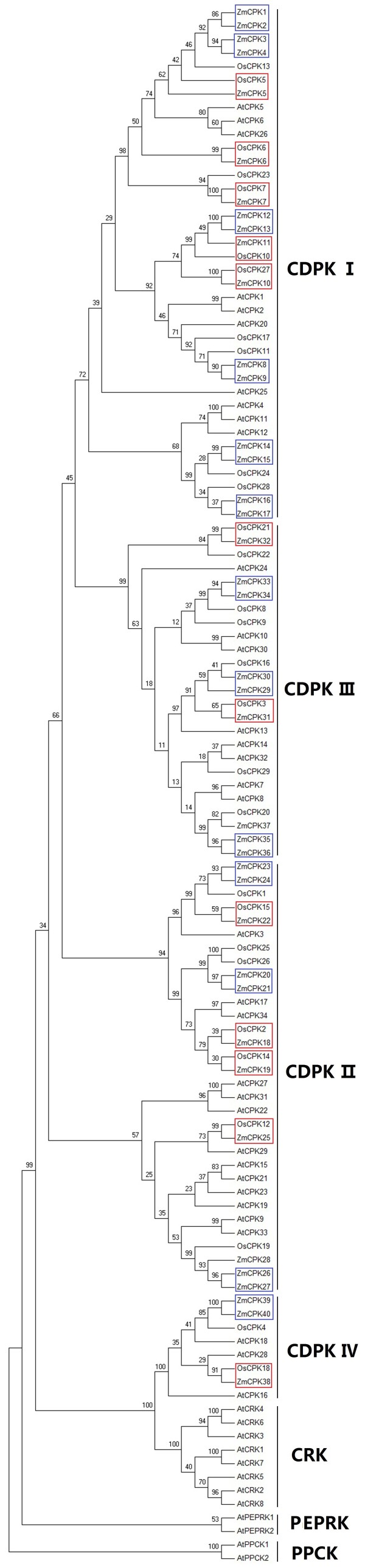
**Phylogenetic tree of CDPKs from maize**, **rice and *Arabidopsis.*** Neighbor-joining tree was created using MEGA5.0 program with 1,000 bootstrap using full length sequences of 40 maize, 29 rice, and 34 *Arabidopsis* CDPK proteins. Four groups were labeled as I, II, III, and IV. Red boxes, maize-rice orthologs; blue boxes, duplicated genes.

It is possible to identify all CDPK gene family members in maize because the maize genome has been completely sequenced [48]. BLAST searches of the maize genome were performed using *Arabidopsis* and rice CDPK sequences as query sequences; this analysis identified 40 putative CDPK genes including 7 known CDPKs, designated as *ZmCPK1*-*ZmCPK40* according to the proposed nomenclature for CDPK genes [34] (Table 1). Because alternative splice variants are closely related to each other based on the results of the multiple sequence alignment and phylogenetic analysis, we selected only a single variant for further analysis. Although the size of maize genome (~2300 Mb) was much larger than the genomes of *Arabidopsis* (125 Mb) and rice (389 Mb), the total number of maize CDPK genes was similar to the number of these genes in *Arabidopsis* and rice. Moreover, the difference in the total number of CDPK genes was mainly due to the expansion of Group I; 17 genes from this group were found in maize, 11 in rice and 10 in *Arabidopsis* (Figure 1).

**Table 1:** Characteristics of CDPKs from maize

**Name (previous name)**	**ID**	**Alternative splicing**	**CDs**	**Amino acid**	**MW (kDa)**	***N*-terminal**	***N*-Myristoylation**	**No. of EF hands**
ZmCPK1	GRMZM2G314396_T01	-	1644	547	604	MGNACSGA	Yes	4
ZmCPK2 (ZmCDPK1)	D84408 ^a^	-	1479	492	547	MRRGGAGA	No	4
ZmCPK3 (ZmCK1)	GRMZM2G321239_T01	-	1671	556	612	MGNACGGA	Yes	4
ZmCPK4 (ZmCDPK7)	D87042 ^b^	-	1665	554	616	MGNACGGA	Yes	4
ZmCPK5	GRMZM2G081310_T01	3	1689	562	619	MGNTCGVT	Yes	4
ZmCPK6	GRMZM2G347047_T01	-	1467	488	539	MGGHQLHL	No	4
ZmCPK7	GRMZM2G032852_T02	3	1635	544	617	MGNQCPNG	No	1
ZmCPK8	GRMZM2G027351_T01	2	1755	584	641	MGNTCVGP	No	4
ZmCPK9	GRMZM2G121228_T01	-	1743	580	637	MGNTCVGP	No	4
ZmCPK10	GRMZM2G353957_T01	-	1941	646	717	MGNVCVGP	No	4
ZmCPK11	GRMZM2G028926_T01	-	1827	608	664	MGNTCVGP	No	4
ZmCPK12	GRMZM2G320506_T01	-	1863	620	677	MGNTCVGP	No	4
ZmCPK13 (ZmCDPK10)	AJ007366 ^c^	-	1920	639	695	MGNTCVGP	No	4
ZmCPK14	GRMZM2G035843_T01	-	1527	508	565	MQPDPSGN	No	4
ZmCPK15 (ZmCDPK11)	GRMZM2G047486_T01	5	1533	510	566	MQPDPSGN	No	4
ZmCPK16	GRMZM2G347226_T01	2	1548	515	568	MQPDPQGS	No	4
ZmCPK17	GRMZM2G463464_T01	2	1548	515	568	MQPDPQGP	No	4
ZmCPK18	GRMZM2G167276_T01	-	1533	510	562	MGNCCPGS	No	4
ZmCPK19	GRMZM2G340224_T01	2	1842	613	675	MRPSVSMI	No	4
ZmCPK20	GRMZM2G365815_T01	-	1659	552	600	MGQCCSKG	Yes	4
ZmCPK21	GRMZM2G472311_T01	-	1746	581	634	MGQCCSKG	Yes	4
ZmCPK22	GRMZM2G058305_T01	3	1620	539	607	MGGRASRH	Yes	4
ZmCPK23	GRMZM2G025387_T01	3	1593	530	599	MGNRASRH	Yes	4
ZmCPK24	GRMZM5G856738_T02	3	1383	460	525	MEDVKATY	No	4
ZmCPK25	GRMZM2G112057_T01	-	1620	539	610	MGNCFTRK	Yes	4
ZmCPK26	GRMZM2G154489_T01	2	1596	531	594	MGQCCSRA	Yes	4
ZmCPK27 (ZmCDPK2)	ZMU28376 ^d^	-	1542	513	581	MVMAILTR	No	4
ZmCPK28 (ZmCDPK9)	GRMZM2G168706_T01	4	1596	531	594	MGQCCSRA	Yes	4
ZmCPK29	GRMZM2G030673_T01	-	1626	541	605	MGNCCRSP	No	3
ZmCPK30	GRMZM2G088361_T01	-	1623	540	602	MGNCCRSP	No	3
ZmCPK31	GRMZM2G311220_T01	3	1611	536	602	MGNCCRSP	No	3
ZmCPK32	GRMZM2G332660_T01	-	1707	568	628	MGGCYSAF	Yes	4
ZmCPK33	AC2100134_FGT014	-	1617	538	604	MGNCCAAP	Yes	4
ZmCPK34	GRMZM2G104125_T01	-	1608	535	603	MGNCCATP	Yes	4
ZmCPK35	AC2338711_FGT003	-	1620	539	608	MGNCCVTP	No	4
ZmCPK36	GRMZM2G028086_T01	2	1620	539	608	MGNCCVTP	No	4
ZmCPK37	GRMZM2G099425_T01	-	1620	539	608	MGNCCVTP	No	4
ZmCPK38	GRMZM2G365035_T01	-	1539	512	575	MGLCSSST	Yes	4
ZmCPK39	GRMZM2G157068_T01	3	1569	522	585	MGACFSSA	Yes	4
ZmCPK40	GRMZM2G053868_T01	3	1569	522	585	MGACFSSA	Yes	4

^a b c and d^ GenBank accession numbers; - represents no alternative splice variants.

All 40 of CDPKs had conserved CDPK domains, including an *N*-terminal variable domain, a protein kinase domain, an autoinhibitory domain, and a calmodulin-like domain. In *Arabidopsis*, rice and wheat, many CDPKs have potential *N*-myristoylation motifs for membrane association at the beginning of their highly variable *N*-terminal domain, with a Gly residue at the second position. Seventeen of the forty maize CDPKs were predicted to have *N*-myristoylation motifs for membrane association. Among them, fifteen CDPKs had at least one Cys residue at positions 3, 4, or 5, which are potential palmitoylation sites (Table 1). In many systems, *N*-myristoylation and palmitoylation promote protein-membrane interactions. AtCPK2, AtCPK10, AtCPK3, TaCPK2 and TaCPK5 were predicted to have an *N*-myristoylation motif and have been shown to be associated with plasma membranes [24,37,49]. In addition, OsCPK19 has been experimentally shown to be myristoylated and palmitoylated and then targeted to the membrane fraction [50]. Although AtCPK5, AtCPK6, TaCPK3 and TaCPK15 lacked myristoylation motifs, these genes were also shown to be associated with the membranes [37]. In maize, both ZmCPK4 and ZmCPK5 had an *N*-myristoylation motif and were predominately localized to the plasma membrane (our unpublished data and See Additional file 1: Figure S1); however, although ZmCK1 (ZmCPK3) was predicted to have an *N*-myristoylation motif, a ZmCK1::hGFP fusion protein was recently found to localize to the cytoplasm and nucleus [51]. These results suggest that other motifs may affect the membrane association of these genes.

In *Arabidopsis*, rice and wheat, most of the calmodulin-like domains of CDPKs contain four Ca^2+^ binding EF hands, allowing the protein to function as a Ca^2+^ sensor. Sequential deletion of the EF hands demonstrated that the number and position of EF hands may be important for determining the Ca^2+^ regulation of CDPK activity. More recently, Franz et al. (2011) showed that *N*-terminal EF1 and EF2 motifs of AtCPK21 were more important for Ca^2+^-regulated enzyme activity when compared to the *C*-terminal EF3 and EF4 motifs and suggested that the EF1 and EF2 motifs may function as a switch for the protein kinase activity mediating abiotic stress signaling [28]. Most maize CDPKs contained four EF hands; ZmCPK29, ZmCPK30 and ZmCPK31 each had three EF hands (Table 1). Interestingly, ZmCPK7 had only one EF hand, which was also found in its homologs, OsCPK7 and AtCPK25 [34,35]. It would be of interest to explore differences in the biological functions of ZmCPK7 and other ZmCPKs.

### Phylogenetic analysis of the CDPK gene family

To study the evolutionary relationships between different CDPK members, an unrooted tree was constructed from alignments of the full CDPK amino acid sequences and the phylogenetic analysis indicated that 40 ZmCPKs can be divided into four groups (Figure 1). It was reported that CRKs, PPCKs and PEPRKs were closely related to the CDPKs. As shown in Figure 1, ZmCPKs, AtCRKs, AtPPCKs and AtPEPRKs were clustered into 7 distinct groups, which indicated that all 40 ZmCPKs actually belonged to the CDPK family. Group I contained 17 CDPKs from maize, 11 from rice and 10 from *Arabidopsis*. ZmCPK1, ZmCPK2, ZmCPK3 and ZmCPK4 showed a high degree of similarity with OsCPK13 (OsCDPK7), which was shown to be associated with cold, drought and salt stress responses [31]. This similarity indicates that ZmCPK1, ZmCPK2, ZmCPK3 and ZmCPK4 might be involved in abiotic stress. Group II contained 11 maize CDPKs, eight rice CDPKs and 13 *Arabidopsis* CDPKs. ZmCPK25 shared 76% similarity with OsCPK12, a positive regulator of salt tolerance and a negative regulator of blast resistance [33]. Group III contained 9 maize CDPKs, eight rice CDPKs and 8 *Arabidopsis* CDPKs. The amino acid sequence identity between ZmCPK32 and OsCPK21 approached 75%. *OsCPK21* was shown to confer salt tolerance in rice [32]. Group IV contained 3 CDPKs from maize, three from rice and 3 from *Arabidopsis*. ZmCPK38 and AtCPK28 shared 75% similarity at the amino acids level. Recently, *AtCPK28* was reported to regulate plant stem elongation and vascular development by altering the expression of NAC transcriptional and gibberellic acid homeostasis regulators [52]. ZmCPK39 showed the highest similarity with OsCPK4, and *OsCPK4* was shown to be transiently activated in *G*. *intraradices*-inoculated rice roots [53].

Phylogenetic analysis showed that there were 14 closely related maize-rice orthologs (ZmCPK5 and OsCPK5, ZmCPK6 and OsCPK6, ZmCPK7 and OsCPK7, ZmCPK10 and OsCPK27, ZmCPK11 and OsCPK10, ZmCPK17 and OsCPK28, ZmCPK18 and OsCPK2, ZmCPK19 and OsCPK14, ZmCPK22 and OsCPK15, ZmCPK25 and OsCPK12, ZmCPK29 and OsCPK16, ZmCPK31 and OsCPK3, ZmCPK32 and OsCPK21, ZmCPK38 and OsCPK18) (Figure 1), suggesting that an ancestral set of CDPK genes existed prior to the maize-rice divergence. Furthermore, most of the maize-rice orthologs had similar *N*-myristoylation motifs, numbers of EF hands and gene structures (See Additional file 2: Figure S2).

### Structural organization of genes and chromosomal localization

**Figure 2:**
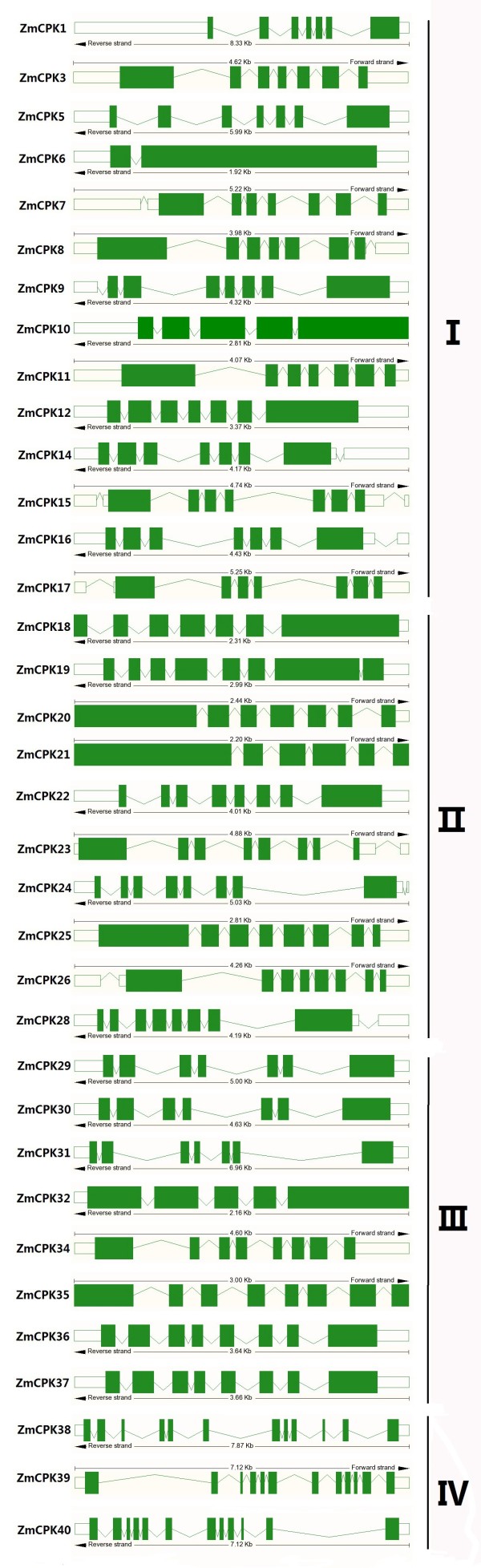
**Exon**–**intron structures of maize *CDPK *genes.** Boxes, exons; green boxes, open reading frames; lines, introns. Four groups were labeled as I, II, III, and IV.

The exon-intron structure of the maize CDPK genes was determined based on the predicted sequences. As shown in Figure 2, most members in the same group had similar exon-intron structure. There were 2–9 exons in maize Group I - III CDPKs, whereas Group IV CDPKs contained 11–12 exons, which is consistent with the exon numbers of *Arabidopsis* and rice CDPKs. This conserved exon-intron structure in each group among all three species supports their close evolutionary relationship and the introduced classification of groups. In addition, our bioinformatics analysis showed that 16 of the ZmCPKs had alternatively spliced mRNAs (Table 1).

**Figure 3:**
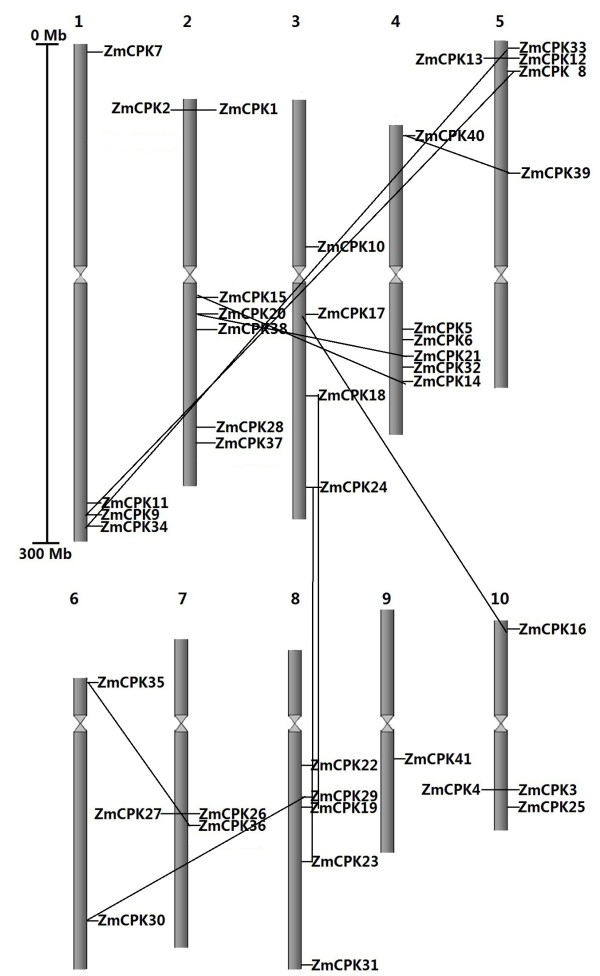
**Chromosomal distributions of *CDPK *genes in the maize genome.** The chromosome number is indicated at the top of each chromosome representation.

*In silico* chromosomal localization of CDPKs indicated that all 40 of the CDPKs were distributed among all 10 chromosomes in maize (Figure 3). In the case of *Arabidopsis* and rice, the 34 and 30 CDPK genes were also distributed among all 5 and 12 chromosomes of their respective genomes, indicating that CDPK genes are widely distributed in plant genomes. However, the distribution of CDPK genes on each maize chromosome was non-random. Several of the CDPKs appeared to be clustered together on specific chromosomes, including chromosomes 1, 2, 4, 5, 7, 8 and 10. In contrast, chromosome 6 had two CDPK genes, whereas chromosome 9 only encoded one CDPK gene.

Several rounds of genome duplication events have been detected in the maize genome. A total of ten segmental duplication events (*ZmCPK8* and *ZmCPK9*, *ZmCPK14* and *ZmCPK15*, *ZmCPK16* and *ZmCPK17*, *ZmCPK18* and *ZmCPK19*, *ZmCPK20* and *ZmCPK21*, *ZmCPK23* and *ZmCPK24*, *ZmCPK29* and *ZmCPK30*, *ZmCPK33* and *ZmCPK34*, *ZmCPK35* and *ZmCPK36*, *ZmCPK39* and *ZmCPK40*) and four gene pairs as tandem repeats (*ZmCPK1* and *ZmCPK2*, *ZmCPK3* and *ZmCPK4*, *ZmCPK12* and *ZmCPK13*, *ZmCPK26* and *ZmCPK27*) were found in the maize genome (See Figures 1, 3 and Additional file 3: Figure S3). These results indicate that both segmental and tandem duplications play an important role in CDPK gene expansion in the maize genome. In *Arabidopsis*, each of the 7 gene paralogs (*AtCPK4* and *AtCPK11*, *AtCPK1* and *AtCPK2*, *AtCPK10* and *AtCPK30*, *AtCPK7* and *AtCPK8*, *AtCPK17* and *AtCPK34*, *AtCPK15* and *AtCPK21*, *AtCPK9* and *AtCPK33*) has the same number of EF hands and *N*-myristoylation motifs. Moreover, both AtCPK4 and AtCPK11 regulate ABA signaling through the phosphorylation of ABF1 and ABF4. In the present study, all of the close paralogs, except ZmCPK1 and ZmCPK2 and ZmCPK26 and ZmCPK27, had similar characteristics, which included *N*-terminal, *N*-myristoylation motifs and the number of EF hands (Table 1). These results suggest that the genes that are close paralogs may also have similar functions.

### Expression pattern of the maize CDPK genes in different tissues and developmental stages

**Figure 4:**
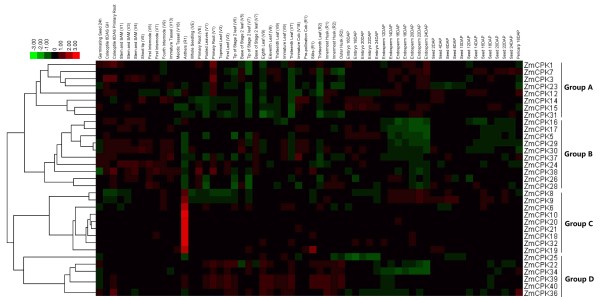
**Expression profiles of maize *CDPK *genes across different tissues and developmental stages.** The scale representing the relative signal intensity values is shown above. DAP: Days After Pollination; DAS: Days After Sowing.

To investigate the expression profiles of CDPK in maize development, we analyzed the expression of the CDPK genes in different tissues and developmental stages using published microarray data. Thirty-three of the 40 maize CDPK genes have the corresponding probe sets in the dataset, and probes for the other 7 CDPK genes were not found. A heatmap representation of the expression profile for 33 CDPK genes during maize development is shown in Figure 4. Based on hierarchical clustering, the expression patterns of the CDPK genes could be divided into four groups: Groups A, B, C and D. Group A CDPK genes had lower expression in leaves than in other organs, whereas Group D CDPK genes were expressed most highly in leaves and primary roots. Interestingly, Group C genes were expressed most highly in anther, suggesting that Group C CDPK genes may play an important role in pollen development. In addition, Group B genes had higher expression in early developmental stages but lower expression in endosperm and seed development, suggesting that Group B CDPK genes may negatively control seed development. Furthermore, CDPK duplicated gene pair expression patterns were also investigated, and most gene pairs (*ZmCPK8* and *ZmCPK9*, *ZmCPK14* and *ZmCPK15*, *ZmCPK16* and *ZmCPK17*, *ZmCPK18* and *ZmCPK19*, *ZmCPK20* and *ZmCPK21*, *ZmCPK29* and *ZmCPK30*, *ZmCPK39* and *ZmCPK40*) shared similar expression patterns in nearly all of the organs and developmental stages analyzed; however, this was not the case for *ZmCPK23* and *ZmCPK24*. These results suggest that most of the CDPK genes could play an important role in maize development.

**Figure 5:**
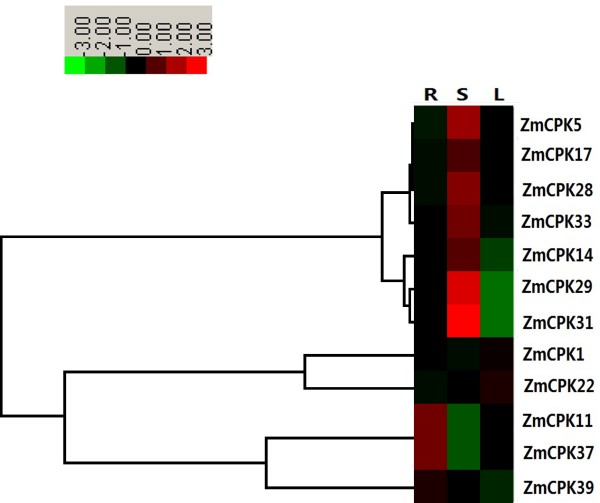
**Tissue**-**specific gene expressions of 12 *CDPK *genes in various tissues by quantitative real**-**time RT**-**PCR analysis.** The scale representing the relative signal intensity values is shown above. Hierarchical clustering was played in data analysis. R: roots; S: stems; L: leaves.

Next, quantitative real-time RT-PCR analysis was performed to examine the CDPK gene expression patterns in roots, leaves and stems. Most of our qRT-PCR data were consistent with the microarray data. *ZmCPK5*, *ZmCPK14*, *ZmCPK17*, *ZmCPK28*, *ZmCPK29*, *ZmCPK31* and *ZmCPK33* were predominantly expressed in stems. For example, *ZmCPK29* and *ZmCPK31* showed a 6-fold and 16-fold increase, respectively, in their expression in stems relative to their expression in roots (Figure 5). *NtCDPK1* is also highly expressed in stems [54]. Three of the 12 ZmCPKs (*ZmCPK11*, *ZmCPK37*, and *ZmCPK39*) were predominantly expressed in roots. In contrast, the *ZmCPK22* transcript levels in leaves were higher than in roots and stems (Figure 5). Intriguingly, four maize-rice orthologs (*ZmCPK11* and *OsCPK10*, *ZmCPK28* and *OsCPK19*, *ZmCPK29* and *OsCPK16*, *ZmCPK33* and *OsCPK8*) exhibited similar tissue-specific expression patterns [55].

### Expression profiles of the maize CDPK genes under abiotic stress

**Figure 6:**
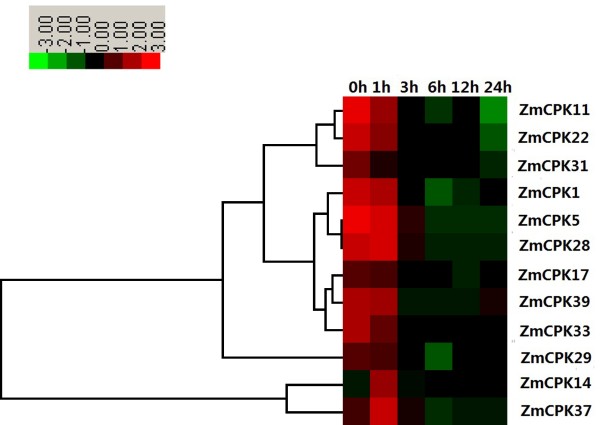
**Expression analysis of 12 *CDPK *genes in roots of maize exposed to 250 mM NaCl for various times as indicated by quantitative real**-**time RT**-**PCR analysis.** The scale representing the relative signal intensity values is shown above. Hierarchical clustering was played in data analysis.

Increasing evidence indicates that CDPKs are involved in various physiological adaptations in response to environmental stimuli, and the expression of CDPK genes are also regulated by various stimuli, including hormones, salt, cold, drought, heat and wounding. In wheat, 10 out of 14 CDPK genes appeared to respond to abiotic stress including drought, NaCl and ABA [36]. To identify the effects of CDPK gene expression on maize stress responses, 14-day-old maize seedlings were treated under conditions of 250 mM NaCl (salt), 20% PEG (drought) and 4°C (cold). We examined the expression levels of 12 maize CDPKs by qRT-PCR. As shown in Figure 6, NaCl treatment caused a marked decrease in the transcription levels of 9 CDPK genes (*ZmCPK1*, *ZmCPK5*, *ZmCPK11*, *ZmCPK17*, *ZmCPK22*, *ZmCPK29*, *ZmCPK31*, *ZmCPK33* and *ZmCPK39*) in roots. The *ZmCPK14* and *ZmCPK37* transcript levels increased 3.5- and 2.9-fold, respectively, at 1 h after NaCl treatment (Figure 6), whereas NaCl slightly up-regulated *ZmCPK28* expression (Figure 6). In rice, eight CDPKs transcripts were down-regulated in response to salt stress. The expression levels of *ZmCPK11*, *ZmCPK29*, *ZmCPK31* and *ZmCPK37* showed a high degree of similarity with the expression levels of their orthologs in rice (*OsCPK10*, *OsCPK16*, *OsCPK3* and *OsCPK20*), which were also down-regulated in response to NaCl treatment [19]. Although multiple studies have reported that many plant CDPKs positively regulate salt/drought stress, our data suggest that these ZmCPKs may negatively control salt stress as well as their OsCPKs homologous genes. However, *ZmCPK4* was down-regulated by NaCl treatment (our unpublished data), whereas the transcripts of its paralogous gene, *ZmCPK3* (*ZmCK1*), accumulated greatly in response to salt stress [51], thus indicating the divergence of CDPK functions of this gene paralogs in maize family. Furthermore, two duplicated CDPK genes in wheat, *CPK7* and *CPK12* displayed the opposite expression patterns in response to abiotic stress or hormone treatments [38]. These results show that although the duplicated genes are highly similar at the amino acid level, they may actually possess different gene functions.

**Figure 7:**
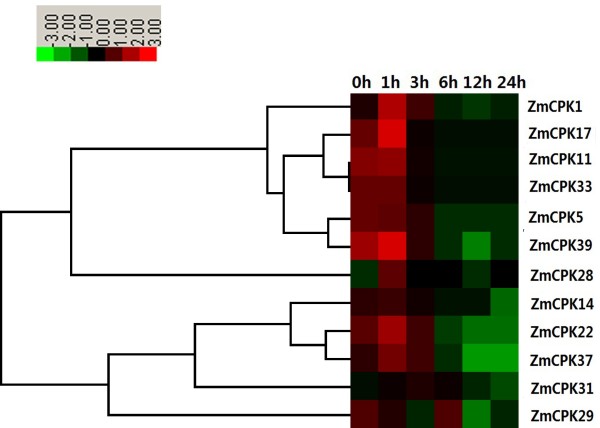
**Expression analysis of 12 *CDPK *genes in roots of maize exposed to 20% PEG for various times as indicated by quantitative real**-**time RT**-**PCR analysis.** The scale representing the relative signal intensity values is shown above. Hierarchical clustering was played in data analysis.

Many CDPK genes identified in various plant species have been proven to play crucial roles during drought stress. Under PEG treatment, *ZmCPK1*, Z*mCPK17*, *ZmCPK22* and *ZmCPK28* were found to be up-regulated between 2.0- and 3.2-fold at 1 h, whereas *ZmCPK11*, *ZmCPK14*, *ZmCPK31*, *ZmCPK37* and *ZmCPK39* were up-regulated between 1.2- and 1.8-fold when compared to the untreated roots (Figure 7). Conversely, two genes, *ZmCPK5* and *ZmCPK33*, were obviously down-regulated after PEG treatment. In addition, *ZmCPK29* had similar expression profiles after PEG treatment, as shown in Figure 7, PEG treatment caused a decrease in transcription levels at 3 h in roots, followed by a quick recovery to untreated levels at 6 h before decreasing again.

**Figure 8:**
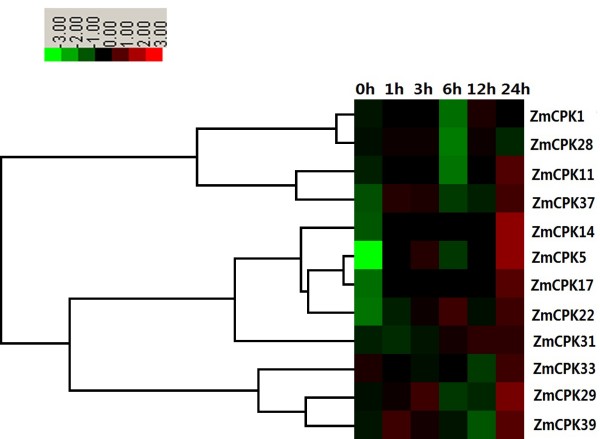
**Expression analysis of 12 *CDPK *genes in roots of maize exposed to 4°C for various times as indicated by quantitative real**-**time RT**-**PCR analysis.** The scale representing the relative signal intensity values is shown above. Hierarchical clustering was played in data analysis.

Ca^2+^-mediated early cold induction of the CBFs is crucial for cold tolerance. In rice, *OsCDPK7* has been shown to enhance cold stress without inducing stress-inducible genes, such as *Rab16A* and *SalT*[31]. The overexpression of *OsCDPK13* also confers cold resistance [56]. In alfalfa, the expression of *MsCK1* and *MsCK2* was induced by cold stress [57]. However, the molecular functions of CDPKs during cold stress signaling remain to be explored. In maize, six genes (*ZmCPK5*, *ZmCPK11*, *ZmCPK22*, *ZmCPK29*, *ZmCPK37*, and *ZmCPK39*) shared similar expression profiles under 4°C treatment conditions. In particular, 4°C treatment conditions induced a biphasic response in which the first peak (phase I) occurred after 1–3 h, and the second peak (phase II) appeared within 24 h (Figure 8). However, there were some differences in the cold-induced expression levels of these ZmCPKs. The cold-induced expression of *ZmCPK11* in phases I and II was slight, and the expression of *ZmCPK5* in phases I and II was the highest among the ZmCPKs genes, which showed more than a 20- and 40-fold change in response to the 4°C treatment conditions, respectively (Figure 8). These results suggest that *ZmCPK5* may play an important role in cold stress and that *ZmCPK5* is a good candidate for developing our understanding the mechanisms of cold tolerance in maize. The 4°C treatment caused a decrease in the transcription levels of *ZmCPK1* and *ZmCPK28* within 6 h, which then increased at 12 h, whereas the transcription of *ZmCPK33* was down-regulated within 12 h but increased at 24 h after 4°C treatment (Figure 8). Under cold conditions, *ZmCPK14*, *ZmCPK17* and *ZmCPK31* transcripts gradually accumulated (Figure 8).

### Expression profiles of the maize CDPK genes in response to exogenous ABA and H_2_O_2_

**Figure 9:**
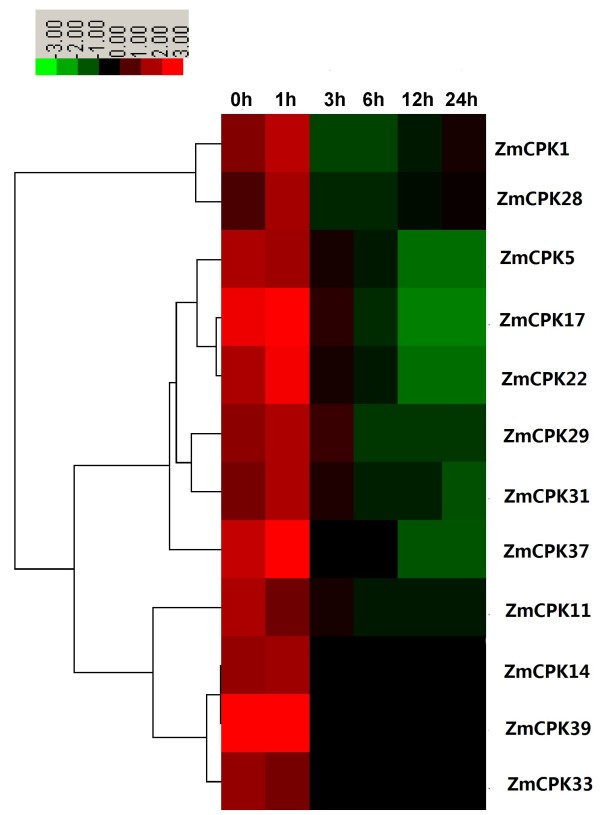
**Expression analysis of 12 *CDPK *genes in roots of maize exposed to 100 μ**M** ABA for various times as indicated by quantitative real**-**time RT**-**PCR analysis.** The scale representing the relative signal intensity values is shown above. Hierarchical clustering was played in data analysis.

The phytohormone abscisic acid (ABA) plays important roles in many aspects of plant growth and development, particular in the physiological response to environmental stressors that include salinity, drought and cold [3]. An increasing body of evidence has shown that CDPKs regulate ABA-mediated signal transduction in plants [22,23,49]. To investigate the possible involvement of CDPKs in ABA- mediated signaling, we examined the expression of 12 CDPK genes in response to ABA treatment. For most of the 12 CDPK genes, ABA treatment led to an increase in transcript levels within 1 h, and then decreased (Figure 9). In contrast, ABA treatment caused a decline in the expression of *ZmCPK5*, *ZmCPK11*, and *ZmCPK33* (Figure 9). In addition, *ZmCPK1* and *ZmCPK39* transcript levels were slightly increased and then quickly decreased in response to ABA treatment (Figure 9). In *Arabidopsis*, *AtCPK4* and *AtCPK11* have been implicated as two important positive regulators in the CDPK-mediated ABA signaling pathway [22]. By contrast, *AtCPK12*, the closest homolog of *AtCPK4*/*AtCPK11*, negatively regulates ABA responses by phosphorylating ABI2, a negative regulator of ABA signaling [58]. In maize, *ZmCPK14*, the homolog of *AtCPK4*/*AtCPK11*, was clearly down-regulated after ABA treatment (Figure 9), suggesting that *ZmCPK14* might act a negative regulator in ABA signaling.

**Figure 10:**
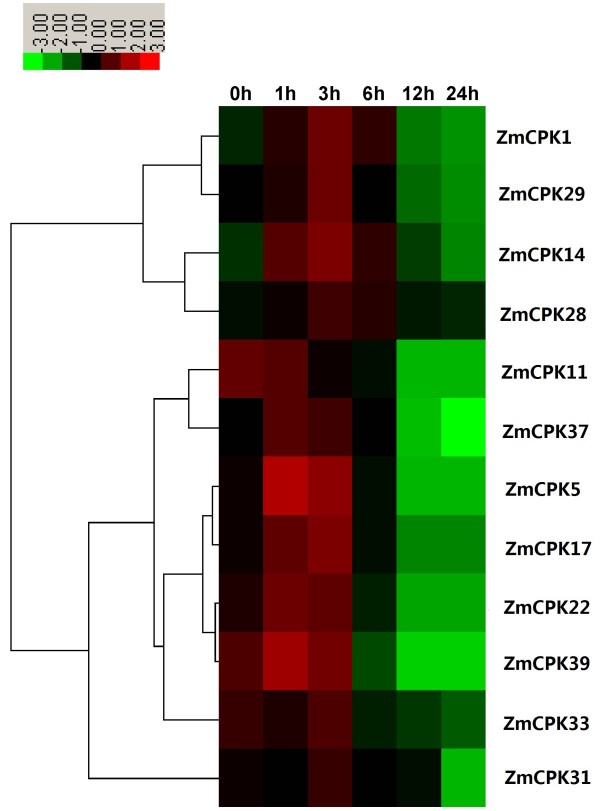
**Expression analysis of 12 *CDPK *genes in roots of maize exposed to10 mM H**_**2**_**O**_**2 **_**for various times as indicated by quantitative real**-**time RT**-**PCR analysis.** The scale representing the relative signal intensity values is shown above. Hierarchical clustering was played in data analysis.

Various environmental and developmental stimuli induce the accumulation of hydrogen peroxide (H_2_O_2_), which acts as a signaling molecule that regulates plant development, stress adaptation, hormone signaling and programmed cell death. In rice, *OsCPK12* regulates ROS homeostasis in response to salt stress by inducing the expression of the ROS scavenger genes *OsAPX2*/*OsAPX8* and by repressing the NADPH oxidase gene *OsRBOHI*, thereby leading to increased salt tolerance [33]. However, *StCDPK4*/*5* induces an oxidative burst by phosphorylating NADPH oxidase [18]. To determine whether maize CDPK genes play a role in H_2_O_2_ signaling pathways, we analyzed the expression of 12 ZmCPK genes in response to H_2_O_2_. The transcript levels of six CDPK genes (*ZmCPK1*, *ZmCPK14*, *ZmCPK17*, *ZmCPK28*, *ZmCPK29*, and *ZmCPK31*) and four CDPK genes (*ZmCPK5*, *ZmCPK22*, *ZmCPK37*, and *ZmCPK39*) were significantly increased at 3 h and 1 h, respectively, after H_2_O_2_ treatment (Figure 10). In response to H_2_O_2_ treatment, the expression of *ZmCPK33* increase slightly and then quickly decreased (Figure 10). The expression of *ZmCPK11* was obviously down-regulated after H_2_O_2_ treatment (Figure 10). These results suggest that maize CDPKs are most likely involved in the H_2_O_2_ signaling pathway.

## Conclusion

In this study, we have conducted a genome-wide analysis of the CDPK gene family, one of the largest protein kinases families in plants, in maize for the first time. *In silico* analysis of the maize genome database resulted in the identification of 40 CDPK genes from maize, and among these genes, 33 were fond to be novel. Our analyses suggest that genome duplications may have contributed to the expansion of ZmCPKs. The phylogenetic analysis of CDPKs from maize, rice and *Arabidopsis* has facilitated the classification of these genes into four groups. Members within each group may have recent common evolutionary origins, as they shared common protein motifs and exon-intron structures. Our microarray analysis showed that most maize CDPK genes exhibited different expression levels in different tissues and at different developmental stages, suggesting that CDPK genes play different roles in maize development. Salinity and oxidative stresses in maize roots have shown a maximum transcript abundance of *ZmCPK14*, and *ZmCPK1*, *ZmCPK5* and *ZmCPK37* demonstrated the highest expression levels in response to drought, cold and ABA, respectively. Our results also suggest that most gene family members may be negative regulators in salt stress signaling pathways. Overall, we have identified a novel CDPK family, and the results presented here provide a solid foundation for further functional investigation of the CDPK gene family in maize.

## Methods

### Database search for CDPK genes in maize

The protein sequences for 34 *Arabidopsis* CDPKs and 30 rice CDPKs were downloaded from the *Arabidopsis* Information Resource (http://www.Arabidopsis.org/) and the rice genome annotation database (http://rice.plantbiology.msu.edu/) or GenBank (http://www.ncbi.nlm.nih.gov/genbank/) respectively. Sequences from the maize genome database were downloaded from (http://www.maizesequence.org/index.html). For the identification of the maize CDPK gene family, *Arabidopsis* and rice CDPKs protein sequences were used to search the maize genome and NCBI database using BLASTP. A self BLAST of the sequences was performed to remove redundancy. All putative candidates were manually verified with the InterProScan program (http://www.ebi.ac.uk/Tools/pfa/iprscan/) to confirm the presence of the protein kinase domain. Finally, all obtained protein sequences were further examined using the Pfam (http://pfam.sanger.ac.uk/search) and SMART (http://smart.embl-heidelberg.de/) resources.

### Phylogenetic analysis of maize CDPK proteins

Multiple alignments of amino acid sequences were aligned using Clustal X. A phylogenetic tree was created according to the neighbor-joining method using the MEGA5.0 program.

### Gene duplication of maize CDPK genes

Gene duplication events of CDPK genes in maize B73 were investigated. We defined the gene duplication using the following criteria: 1) the alignment length covered >80% of the longer gene, 2) the aligned region had an identity >80% and 3) only one duplication event was counted for tightly linked genes. All of the relevant genes identified in the maize genomes were aligned using Clustal X and then calculated using MEGA v5.0.

### Plant materials and growth conditions

Maize seedlings (*Zea mays* L. cv Zhengdan 958) were grown in Hoagland’s solution (pH 6.0) under greenhouse conditions at 25°C/22°C (day/night) with a photosynthetic active radiation of 200 μmol m^-2^ s^-1^ and a photoperiod of 16/8 h (day/night) for 2 weeks.

### Stress treatments

The 2-week-old maize seedlings were dipped in Hoagland’s solution containing 250 mM NaCl, 20% PEG6000 (w/v), 100 μ**M** ABA, 10 mM H_2_O_2_ at 25°C/22°C (day/night) with a continuous light intensity of 200 μmol m^-2^ s^-1^. A low temperature treatment was carried out at 4°C under the same light periods, and plants were watered with Hoagland’s solution. Samples were collected at different intervals after treatments and were immediately frozen in liquid N_2_ for further use.

### RNA isolation and real-time quantitative RT-PCR expression analysis

Total RNA was extracted from the roots of maize seedlings (following various treatments) using Trizol reagent and according to the manufacturer’s instructions (Invitrogen, Carlsbad, CA, USA). First strand cDNAs were synthesized using the First Strand cDNA Synthesis kit (Fermentas, USA).

Real-time quantification RT-PCR reactions were performed in the Bio-RAD MyiQ^TM^ Real-time PCR Detection System (Bio-Rad, USA) using the TransStart Top Green qPCR SuperMix (TransGen, China) and according to the manufacturer’s instructions. Each PCR reaction (20 μl) contained 10 μl of 2 × real-time PCR Mix (containing SYBR Green I), 0.5 μl of each primer and the appropriately diluted cDNA. The thermal cycling conditions were 95°C for 30 s followed by 45 cycles of 95°C for 15 s, 55°C -60°C for 30 s and 72°C for 15 s. The *Zmactin* gene was used as internal reference for all the qRT–PCR analyses. Each treatment was conducted independently and in triplicate. Relative gene expression was calculated according to the delta-delta Ct method of the system. The primers used are described in Additional file 4: Table S1.

### Microarray analysis

The microarray data for various tissues/organs and developmental stages were obtained from the Maize eFP database (http://bar.utoronto.ca/efp_maize/cgi-bin/efpWeb.cgi) [59,60] using the identified ZmCPK ID (Table 1). The expression profiles were clustered using Cluster 3.0 with Euclidean distances and the hierarchical cluster method of complete linkage clustering.

## Competing interests

The authors have declared that no competing interests.

## Authors' contributions

XK carried out all the experiments and data analyses. WL carried data analyses. XK, WL and DL designed the experiments and wrote the manuscript. SJ, DZ and GC performed the qRT-PCR experiments. JP wrote the manuscript. All authors read and approved the final manuscript.

## Supplementary Material

Additional file 1: Figure S1 Subcellular localization of the ZmCPK5:GFP fusion protein in onion epidermal cells. The cells with constructs expressing GFP alone and the ZmCPK5:GFP fusion protein were analyzed under bright and fluorescence field after 16 h. Click here for file

Additional file 2: Figure S2 Exon–intron structures of maize-rice orthologs genes. Green boxes, exons; lines, introns; Blue boxes, UTR; 0,1,2, intron phase. Click here for file

Additional file 3: Figure S3 Phylogenetic tree of maize CDPKs. Neighbor-joining tree was created using MEGA5.0 program with 1,000 bootstrap using full length sequences of 40 maize. Four groups were labeled as I, II, III, and IV. Click here for file

Additional file 4: Table S1 PCR primers used in this study. Click here for file
