# Supplementary material for: Genome-wide identification and expression analysis of calcium-dependent protein kinase in maize
Source: BMC Genomics. 2013 Jul 1;14:433. doi: 10.1186/1471-2164-14-433 (PMC3704972; doi:10.1186/1471-2164-14-433)

**Figure S1** Subcellular localization of the ZmCPK5:GFP fusion protein in onion epidermal cells. The cells with constructs expressing GFP alone and the ZmCPK5:GFP fusion protein were analyzed under bright and fluorescence field after 16 h.


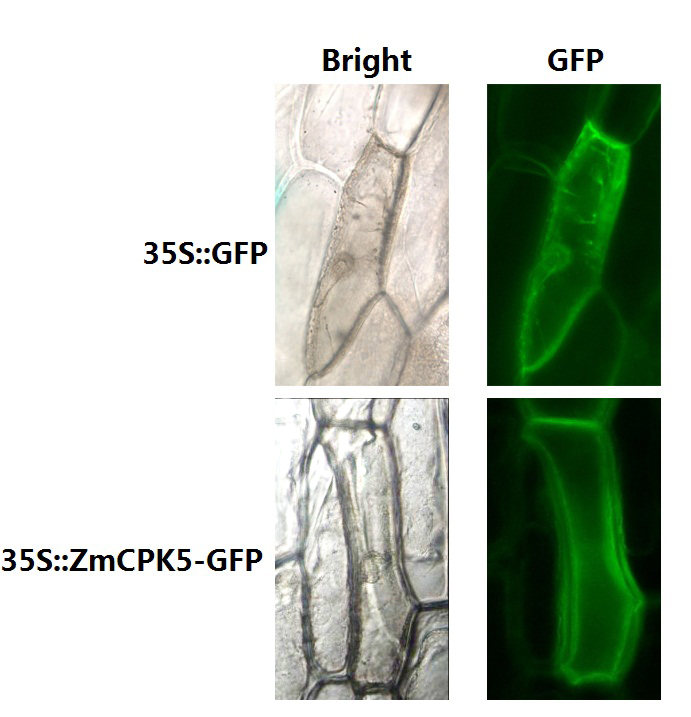

Supplement: Additional file 1: Figure S1 — Subcellular localization of the ZmCPK5:GFP fusion protein in onion epidermal cells. The cells with constructs expressing GFP alone and the ZmCPK5:GFP fusion protein were analyzed under bright and fluorescence field after 16 h. [file 1471-2164-14-433-S1.doc]
