# Supplementary material for: Genome-wide identification and expression analysis of calcium-dependent protein kinase in maize
Source: BMC Genomics. 2013 Jul 1;14:433. doi: 10.1186/1471-2164-14-433 (PMC3704972; doi:10.1186/1471-2164-14-433)

**Figure S2** Exon–intron structures of maize-rice orthologs genes. Green boxes, exons; lines, introns; Blue boxes, UTR; 0,1,2, intron phase.


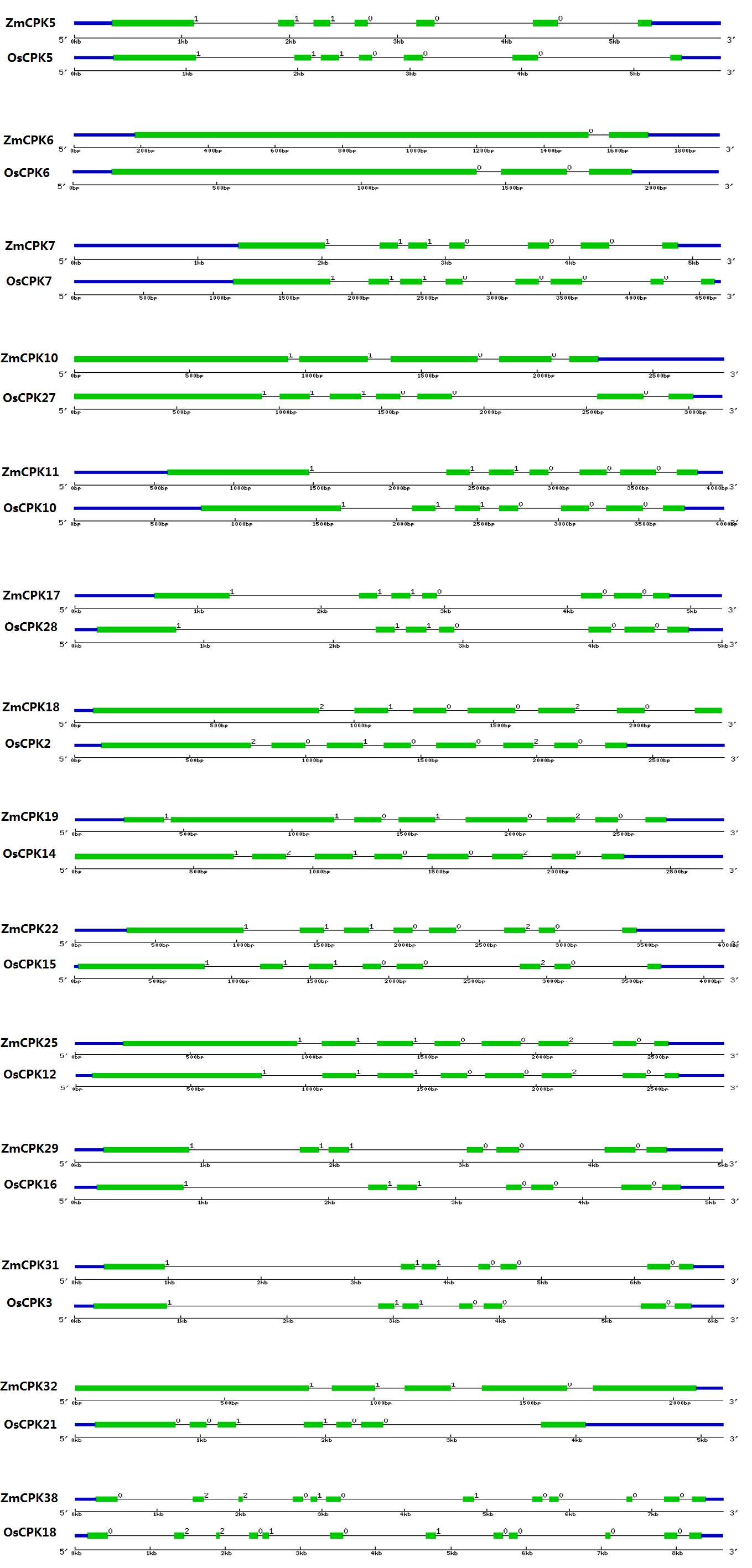

Supplement: Additional file 2: Figure S2 — Exon–intron structures of maize-rice orthologs genes. Green boxes, exons; lines, introns; Blue boxes, UTR; 0,1,2, intron phase. [file 1471-2164-14-433-S2.doc]
