# Supplementary material for: Genome-wide identification and expression analysis of calcium-dependent protein kinase in maize
Source: BMC Genomics. 2013 Jul 1;14:433. doi: 10.1186/1471-2164-14-433 (PMC3704972; doi:10.1186/1471-2164-14-433)

**Figure S3** Phylogenetic tree of maize CDPKs. Neighbor-joining tree was created using MEGA5.0 program with 1,000 bootstrap using full length sequences of 40 maize. Four groups were labeled as I, II, III, and IV.


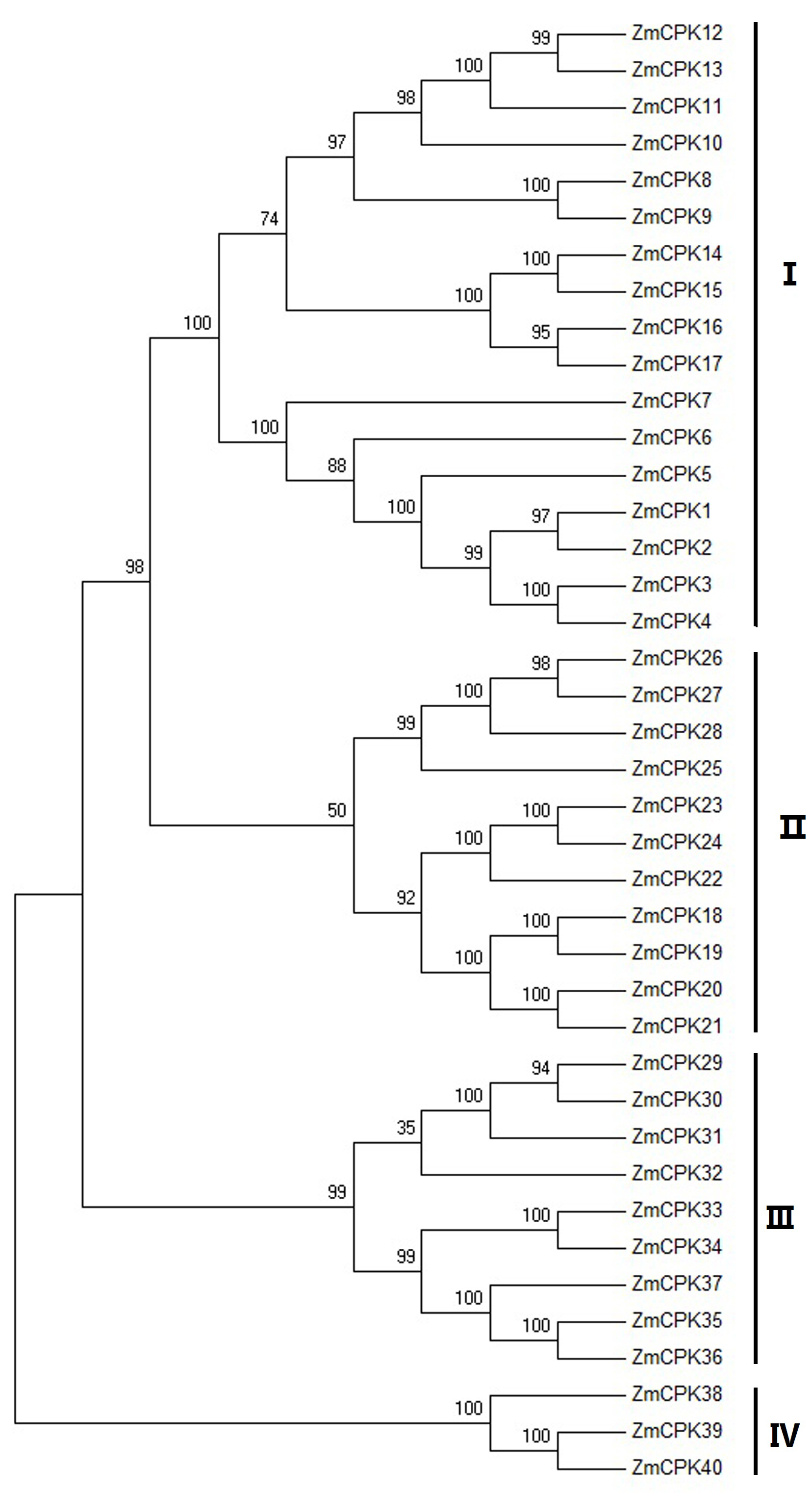

Supplement: Additional file 3: Figure S3 — Phylogenetic tree of maize CDPKs. Neighbor-joining tree was created using MEGA5.0 program with 1,000 bootstrap using full length sequences of 40 maize. Four groups were labeled as I, II, III, and IV. [file 1471-2164-14-433-S3.doc]
