# Supplementary material for: Genome-wide identification and expression analysis of calcium-dependent protein kinase in maize
Source: BMC Genomics. 2013 Jul 1;14:433. doi: 10.1186/1471-2164-14-433 (PMC3704972; doi:10.1186/1471-2164-14-433)
Supplement: Additional file 4: Table S1 — PCR primers used in this study. [file 1471-2164-14-433-S4.doc]

**Table S1** PCR primers used in this study

| Gene | Forward primer | Reverse primer |
| --- | --- | --- |
| *ZmCPK1* | 5'-GTGTCGTAGGCGGTTTGC-3' | 5'-CGCTGTACTCGGAGTCGTG-3' |
| *ZmCPK5* | 5'-TGCACCTCAATAAACTGGAACG-3' | 5'-GCCCATCATTATCCTGGTCAGC-3' |
| *ZmCPK11* | 5'-AAGGATCTTGTGAGGAGAATGC-3' | 5'-CTTGTCAGGAGCCAAACCAC-3' |
| *ZmCPK14* | 5'-ACTTGGTTTCGGCATTCTCATT-3' | 5'-CCTCATCATCGCTGTGAACTCG-3' |
| *ZmCPK17* | 5'-TTTAGTCCGTAAGATGCTTATCCG-3' | 5'-AATAGGCTTATCAGGTGCGACA-3' |
| *ZmCPK22* | 5'-ATGAGGTGTGTCGTGTGGTCTA-3' | 5'-GCATTACTTTTCGCCTGGTAT-3' |
| *ZmCPK28* | 5'-CGTCCTCCCTCCAACTCCAAGA-3' | 5'-TGCGTCGATGAGACAAATCAAGAGC-3' |
| *ZmCPK29* | 5'-TTTGTCTGGGATTTAGGTAGTT-3' | 5'-AAACATTTTACAACACTTCCATT-3' |
| *ZmCPK31* | 5'-GTGACTGTCTTGCGTGGAAAT-3' | 5'-AGCATTAGGTACACCGTTTGG-3' |
| *ZmCPK33* | 5'-GTGTAGTGGTGGTGCTGGTGTT-3' | 5'-CGTGGAGGTGGAATCTAATGAC-3' |
| *ZmCPK37* | 5'-TGTTGATGCCGTGATTGTATTT-3' | 5'-ATGGGTCCACAGAAGTTAAGAA-3' |
| *ZmCPK39* | 5'-TCTTTGGGTTCTTCAGAGTGC-3' | 5'-AGGCGAGTCTTGCCTACCATT-3' |
| *Zmactin* | 5'-ATCCAGGCTGTTCTTTCGTT-3' | 5'-CATTAGGTGGTCGGTGAGGT-3' |
